# Intersecting Roles of Estrogens and Neutrophils in Modulating Innate Immunity in Cancer

**DOI:** 10.3390/biom16050617

**Published:** 2026-04-22

**Authors:** Mary Wines-Samuelson, Thomas R. Henson, Raegan J. Myers, Stephen R. Hammes

**Affiliations:** 1 Wegmans School of Pharmacy, St. John Fisher University, Rochester, NY 14618, USA; 2 Department of Microbiology and Immunology, Division of Endocrinology and Metabolism, Department of Medicine, University of Rochester Medical Center, Rochester, NY 14642, USA; thomas_henson@urmc.rochester.edu (T.R.H.); raegan_myers@urmc.rochester.edu (R.J.M.); stephen_hammes@urmc.rochester.edu (S.R.H.); 3 Department of Medicine, Division of Endocrinology and Metabolism, University of Rochester Medical Center, Rochester, NY 14642, USA

**Keywords:** innate immunity, cancer, neutrophil, NETs, NETosis, steroid hormone, estrogen

## Abstract

Steroid-sensitive cancers (e.g., breast, ovarian, uterine, and prostate cancers) are difficult to control and frequently metastasize to lymph nodes, bone, or lung. Although endocrine research has greatly advanced our identification of the direct roles of steroid sex hormones such as androgens and estrogens on tumor cells in promoting metastasis or recurrence (e.g., treatment with gonadotropin releasing hormone agonists/antagonists, aromatase inhibitors, and estrogen and androgen receptor antagonists), mechanistic insight regarding indirect effects of steroid hormones, including how the innate immune system responds to cancer and is influenced by steroid hormones, is lacking. Despite technological advances in engineering more robust adaptive immunity to combat tumor growth (e.g., CART or checkpoint inhibitors), there remains a relative lack of investigation into the role of innate immunity as a key defense system. Here we discuss recent studies that highlight the significance of neutrophils and their response to tumorigenic conditions with or without steroid hormones in animal models of cancer. We will describe relationships between steroid hormones and neutrophils, with a specific focus on neutrophil extracellular traps (NETs), and how these interactions modulate tumor growth and invasion. Together, these data indicate that combinatorial regulation of both innate and adaptive immunity in the context of tumorigenesis may improve outcomes in cancer therapies.

## 1. Introduction

The presence and function of neutrophils in the tumor microenvironment play critical roles in cancer. Neutrophil-derived enzymes and other cellular contents alter the ability of tumors to grow and metastasize [1,2,3]. Metabolic changes and surface expression of checkpoint inhibition markers in neutrophils also influence anti-tumor adaptive immune responses [4,5]. Neutrophils are generally recruited to the tumor microenvironment along with other myeloid derived cells [6,7]. Early cancer responses by these cells are anti-tumoral; however, as tumor establishment progresses, neutrophils become polarized towards alternate functions by the inflammatory tumor secretomes [8,9]. An essential feature of neutrophils for pathogen defense, the formation of extracellular traps (or NETs) in a process termed NETosis becomes activated in the context of cancer and contributes to cancer progression by promoting metastasis and thromboembolus formation, matrix remodeling, and suppression of adaptive immunity [10,11,12,13]. Tumor-derived factors in combination with steroid hormones can shift both myeloid and adaptive immune-derived support towards tumor survival and metastasis [14,15]. This has led generally to an acceptance of neutrophils being associated with poor outcomes in cancer [16,17,18].

Characterizing the roles of neutrophils and identifying methods to manipulate their functions may improve adaptive immune responses against cancer. In this review article, we will attempt to highlight work regarding neutrophils, hormones, and steroid-sensitive tumors and cancers, focusing on estradiol-sensitive cancers like breast cancer and lymphangioleiomyomatosis (LAM). We will discuss the conflicting evidence regarding impacts of steroid hormones on neutrophil function in steroid-sensitive cancers. Finally, we will highlight efforts to suppress neutrophil activation in these diseases, and how anti-neutrophil activation therapies may apply to estrogen-sensitive cancers.

## 2. Estrogens and Their Receptors

Estrogens are essential steroid hormones synthesized from cholesterol that are known to play vital roles in reproduction, bone health, memory, and mood, as well as in inflammation and cancer [19,20,21,22]. Four types of estrogen occur naturally in mammals: estrone (E1), the main estrogen after menopause synthesized by adipocytes in women; estradiol (E2), the most potent estrogen synthesized by ovaries and abundant during the reproductive period; estriol (E3), made by the placenta and present during pregnancy; and estretol (E4), synthesized in utero by the fetus [19]. Males also have estrogen steroids created by aromatase-dependent conversion from androgens to estrogens, the most abundant being estradiol, derived from testosterone. Males have smaller amounts of estrone derived from androstenedione, and estriol, the weakest of the three estrogens in men [23,24].

Estradiol signaling is mediated primarily through intracellular estrogen receptors, ERα, and ERβ, and possibly via membrane-bound G-protein-coupled receptor 1 (GPER1), all of which are expressed on innate immune cells like monocytes, macrophages, and neutrophils [25]. ERα is the most widely expressed estrogen receptor and is the dominant receptor in immune cells [25]. Recent identification of variant forms of ERα (ERα-36, ERα-46 vs. ERα-66) point to distinct roles for the variants in promoting cell proliferation, immune responses, and crosstalk between signaling pathways [26,27].

## 3. Sex-Specific Effects of Estradiol on Innate Immunity

Interestingly, evidence suggests that estradiol regulates the innate immune system. Estradiol function is likely to intersect at multiple levels of the innate immune cell life cycle: control of progenitor fate in the bone marrow, phenotype of the mature immune cells, and survival vs. death. Evaluation of hematopoietic stem cells (HSCs) and common myeloid progenitors (CMPs) in total bone marrow exposed to estradiol and growth-promoting factors revealed enhanced numbers of progenitors, and increased dendritic cells [28,29,30,31]. In another study involving both mice and humans, estradiol was shown to promote increased differentiation of neutrophils from bone marrow progenitors [32]. Finally, estradiol and other selective estrogen receptor modulators have been shown to promote myeloid-derived suppressor cell (MDSC) production in human bone marrow from breast cancer patients [33].

## 4. Neutrophil-to-Lymphocyte Ratio and Sex Bias

A downstream effect of estradiol-mediated neutrophil production from bone marrow, along with estradiol’s ability to enhance neutrophil survival in the periphery, is an overall increase in neutrophil number relative to lymphocyte (B cell, T cell, and natural killer (NK) cell) number. The neutrophil-to-lymphocyte ratio (NLR), a diagnostic measure derived from circulating blood counts, reflects the balance between neutrophil-driven inflammation (innate immunity) and lymphocyte-mediated immune surveillance (adaptive immunity). Consistent with the pro-neutrophil effects of estradiol, women generally exhibit higher NLR values compared to men. After menopause occurs and estradiol decreases, women have fewer circulating neutrophils than younger women and thus a lower NLR, a shift attributed to hormonal changes [34,35], further implicating estrogen as a positive factor in neutrophil production and survival.

Importantly, elevated NLR values have been strongly correlated with poor outcomes in cancer prognosis [36]. A higher ratio indicates a pro-tumorigenic inflammatory state (high neutrophils) versus a weakened anti-tumor immune response (low lymphocytes). This association has been observed across several estrogen-related cancers, including triple-negative breast cancer and cervical cancer [37,38,39,40]. Together, these data strongly indicate that aggressive tumors enhance neutrophil production and survival, which in turn may then enhance tumor progression.

In addition to favoring neutrophil (myeloid) vs. lymphocyte (lymphoid) differentiation in women, estradiol may also enhance neutrophil survival by modulating apoptotic responses. Neutrophils are produced rapidly with short half-lives (6–12 h) to allow for fast surveillance and clearance. Apoptosis, through spontaneous means or Fas-ligand-dependent interactions, aids in neutrophil clearance. Inflammatory recruitment of neutrophils is known to extend the lifespan of neutrophils through various signaling pathways including JAK/STAT3, NF-kB, and PI3K/AKT signaling [41,42,43]. Signaling through estrogen and progesterone receptors may also regulate this via delayed apoptosis, which in tandem with tumor-derived pro-inflammatory factors could collectively influence neutrophil survival and function in steroid-sensitive cancers.

In women, spontaneous neutrophil apoptosis is reduced compared with men [44]. Interestingly, physiologic doses of estradiol and progesterone administered to both men and women caused delays in spontaneous neutrophil apoptosis in both men and women, but did not diminish Fas antibody-induced apoptosis [44]. Moreover, blockade of progesterone or estrogen receptors with selective antagonists precluded the enhanced survival of neutrophils isolated from women. Decreased apoptosis was due to attenuated release of cytochrome c from mitochondria, preventing assembly of the apoptosome and caspase activation; thus, signaling via estrogen receptors on neutrophils prolongs survival by blocking cytochrome c release from mitochondria in the presence of estradiol [44]. This delay in neutrophil apoptosis mediated by estradiol and progesterone is an example of external environmental factors altering important phenotypes of neutrophils in diseases like cancer. Although it may seem beneficial, prolonged neutrophil half-life in the context of disease and specifically cancer can allow education of the neutrophils in a harmful (tumor) context, converting them from anti-tumorigenic to pro-tumorigenic.

## 5. Estrogen-Sensitive Cancers

**Figure 1:**
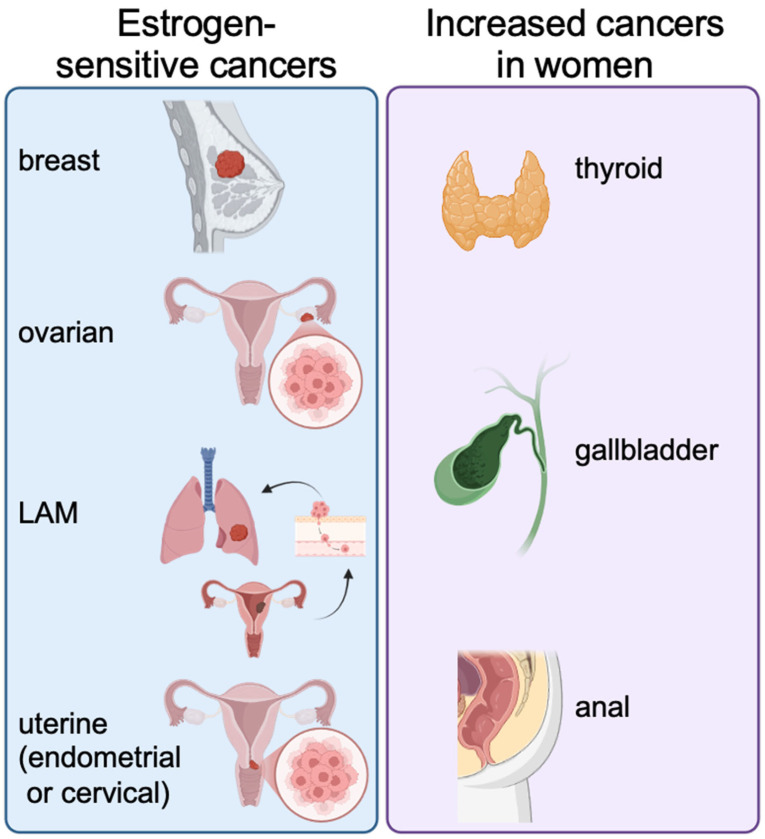
Estradiol-dependent and female-biased cancers. (**Left panel**) In females, estrogen promotes growth of tumors in breast, ovarian, lymphangioleiomyomatosis (LAM), and uterine cancers. Males may develop breast cancer as well, but at much lower levels compared to females. (**Right panel**) Women also experience increased risk of thyroid, gallbladder, and anal cancers, despite the fact that these organs are not considered estrogen-responsive.

Given the links between estradiol, neutrophils, and cancer outcomes, it is reasonable to question whether estrogen-sensitive cancers may be responding both directly to estradiol as well as indirectly through estradiol effects on neutrophils. The most common female-biased cancers are those known as estrogen-dependent (or hormone receptor-positive) cancers [45]. These cancers grow and potentially spread when estradiol activates estrogen receptors (ER) in the cancerous cells [46,47,48]. The primary estrogen-dependent cancers are breast cancer, ovarian cancer, and uterine (endometrial) cancer (Figure 1). Breast cancer is the most prevalent cancer in women worldwide, with ~70–80% of breast cancers being hormone receptor-positive (either estrogen receptor alpha or progesterone receptor) [45]. When estradiol binds to ERα, it stimulates the cell to divide. While breast, ovarian, and uterine cancers are directly linked to estrogen receptors, several other cancers have a higher incidence rate in women compared to men: thyroid cancer, gallbladder cancer, and anal cancer [49] (Figure 1).

Cancer of the endometrium, or the inner-most lining of the uterus, is strongly linked to an imbalance of progesterone, which normally opposes estrogen (Figure 1). Progesterone decreases estrogen receptor expression and can reduce circulating estradiol levels. A risk factor for endometrial cancer is long-term use of estrogen-only hormone therapy, or conditions that lead to excessive production of estradiol (e.g., obesity) [47,50]. Ovarian cancers are more rare than endometrial or breast cancer, with a subset of these being ER-positive and promoted by estrogen (Figure 1) [51].

A unique estrogen-dependent cancer is lymphangioleiomyomatosis (LAM), a rare genetic cancer that exclusively affects women during their childbearing years (Figure 1) [52,53]. It is caused by mutations in the tuberous sclerosis genes *TSC1* or *TSC2*, which lead to constitutive activation of the mTORC1 pathway that then drives cell proliferation. LAM is characterized by a myriad of small smooth muscle-cell-like tumors that in turn promote progressive lung destruction and replacement of normal alveoli with large cysts that eventually progress to significantly impair normal lung function [54,55]. Although the main target organ in LAM patients is the lung, patients also have abnormal smooth muscle-like cells spread through the kidneys and lymphatic system as well, forming benign angiomyolipomas (AMLs) and other tumors [54,55].

While the origin of the LAM cell is unclear, some work suggests that the lung smooth muscle cell tumors may metastasize from the myometrium [32,52]. This myometrium-to-LAM connection is consistent with observations that LAM growth is promoted by estrogen, as LAM is diagnosed after menarche, usually worsens with pregnancy, and slows in progression and symptoms after menopause. That said, the most common treatment for LAM is sirolimus (rapamycin), an FDA-approved drug that inhibits mTOR, the pathway that is upregulated due to loss of TSC1 or TSC2. Of note, as with other steroid-dependent cancers, in patients with LAM, higher NLR values are associated with more aggressive disease, as characterized by worse lung function and increased susceptibility to pneumothorax, key features of LAM disease pathology [56].

## 6. Neutrophils Promote Cancer Cell Proliferation and Migration

As mentioned, neutrophil production and levels in the blood are increased in the setting of many different cancers, but are these increased levels simply a response to the cancer, or are the neutrophils playing a role in promoting cancer progression? In fact, neutrophil-derived factors such as neutrophil elastase (NE) have been shown to impact cancer proliferation. Neutrophil elastase is one of several serine proteases (including cathepsin G and proteinases (PR) 3 and 4) stored in cytoplasmic granules and secreted by neutrophils that cleaves elastin and collagen, which is crucial for fighting pathogen infections [57,58]. However, chronic neutrophil-derived neutrophil elastase in the context of chronic disease (e.g., in cystic fibrosis or chronic obstructive pulmonary disease (COPD)) can cause serious tissue damage [59,60].

The relationship between neutrophil elastase and steroid-sensitive cancers is complex. Interestingly, neutrophil elastase was identified as an estrogen-sensitive gene upregulated in a uterine-specific TSC2-knockout model of lymphangioleiomyomatosis (LAM) [53]. Neutrophil elastase was first shown to increase proliferation of cultured HER2+ breast cancer cells in a dose-dependent manner that was blocked by sivelestat exposure [61]. In the mouse model of LAM, neutrophils were elevated in blood and in the uteri of TSC2-null mice, with markedly increased levels of neutrophil elastase in tumors [62]. Each methodology of neutrophil manipulation explored, including neutrophil depletion, inhibition of recruitment to the tumor bed, and neutrophil elastase inhibition using sivelestat, reduced tumor size in the model [62]. Further, neutrophil depletion attenuated estradiol-mediated lung colonization of tail vein-injected TSC2-null cells [32]. Finally, when TSC2-null LAM cells were cultured in vitro with purified neutrophil elastase, they demonstrated increased migratory capacity that was diminished by treatment with the neutrophil elastase inhibitor sivelestat [62].

While the exact mechanism by which NE modulated tumor cell processes is unclear, studies in prostate cancer cells and in prostate cancer mouse models have revealed some interesting possibilities. First, as with the mouse LAM model, neutrophil depletion similarly reduces prostate cancer cell xenograft growth in immunocompromised mice, and neutrophil elastase inhibition reduces prostate growth in a prostate-specific PTEN-null mouse model [63,64]. Second, expression of the endogenous neutrophil elastase inhibitory protein SERPINB1 in human prostate cancer samples is inversely proportional to tumor aggressiveness (i.e., the more aggressive the tumor, the lower the SERPINB1 expression, and therefore possibly the more the neutrophil elastase activity) [64]. Finally, in vitro studies using purified neutrophil elastase demonstrate that neutrophil elastase promoted tumor cell migration via neutrophil elastase-mediated cleavage of amphiregulin followed by EGFR activation, as well as neutrophil elastase-mediated release of GAS6 followed by AXL activation [65]. Importantly, neutrophil elastase-mediated effects on migration could be a contributing factor to the metastatic nature of LAM and prostate cancer cells, perhaps through the process of neutrophil extracellular trap formation (NETosis)-mediated epithelial–mesenchymal transition (EMT) in tumor cells (see below).

## 7. Estradiol-Driven Recruitment and Polarization of Neutrophils in Tumors

**Figure 2:**
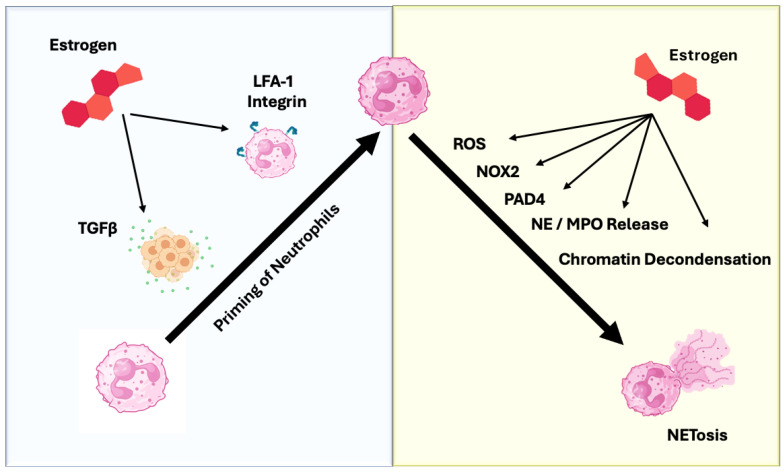
Estrogen-dependent priming and activation of neutrophils in NETosis. Schematic diagram of a neutrophil (pink) and exposure to estradiol, the most potent form of estrogen (red). In patients with tumors and estradiol, neutrophils will adopt a pre-NETosis-primed state. Estradiol-induced expression of both the cytokine TGF-β and the cell surface integrin lymphocyte function-associated antigen (LFA-1) act together to enhance migration and entry into tissue. Once the primed neutrophil enters a target site, it will undergo vital NETosis by increasing ROS production (mainly by NOX2), increasing PAD4 activation and subsequent chromatin decondensation, and promoting degranulation to release neutrophil elastase (NE) and MPO. This vital NETosis then stimulates proliferation, EMT, migration, and invasion of tumor cells, ultimately promoting tumor progression.

While estradiol increases the number of neutrophils circulating in blood, evidence suggests that estradiol also has direct effects on neutrophil function. Estradiol modulates both neutrophil recruitment and polarization; when combined with tumor-derived factors, it shifts neutrophil function from what is generally considered to be anti-tumorigenic (N1) to a pro-tumorigenic (N2) state. Estradiol has been shown to promote recruitment of neutrophils to estrogen receptor (ER)-positive mouse tumors [66]. In addition to neutrophil chemotaxis, estradiol signaling perpetuates the estradiol polarization of neutrophils by increasing the secretion of TGF-β from ER+ tumor cells [66]. Both recruitment and polarization may be occurring primarily through TGF-β, since the same molecule can perform both tasks. Lymphocyte function-associated antigen 1 (LFA-1) integrin may indirectly facilitate “priming” of neutrophils to the N2 state in the context of cancer by promoting firm adhesion in rolling neutrophils and helping them cross the endothelial boundary of blood vessels to invade the tumor itself, where they can be exposed to TGF-β, thus polarizing to N2 [67]. Consistent with the ability of estradiol to promote both recruitment and polarization to tumors, N2-polarized neutrophils have been shown to predominate in multiple estrogen-sensitive tumor types (ovarian, breast) and in highly aggressive tumors in general, relative to their N1-polarized counterparts [68,69,70] (Figure 2).

## 8. Estradiol and NETosis

How does NE, an intracellular protease released from neutrophils, promote tumor progression? Aside from degranulation, NE is released during a process called NETosis. In addition to modulating neutrophil differentiation (i.e., N1 versus N2 neutrophils), estradiol may also play an important role in promoting NETosis, the process by which a neutrophil releases a sticky net of genomic or mitochondrial DNA and pro-oxidative enzymes that can then trap and kill nearby pathogens and/or dysfunctional cells. In fact, one important enzyme released during the NETosis process is NE. Neutrophils undergo NETosis in response to disruptions in homeostasis (e.g., in pro-inflammatory conditions) as seen with cancer or autoimmune disease. Under these inflammatory conditions, potent triggers for NETosis include the inflammatory cytokines interleukin-8 (IL-8) and tumor necrosis factor alpha (TNFα), or crystals (cholesterol or urate). NETosis plays a crucial role in the immune system, where it serves as an essential component of innate immunity by immobilizing and killing pathogens, thereby preventing their spread. However, when NET formation becomes excessive or dysregulated, NET structures can act as a source of damage-associated molecular patterns (DAMPs), promoting excessive inflammation and contributing to tissue injury with the release of destructive proteases. In the context of chronic inflammatory disease, potent triggers for neutrophil NET formation accumulate [71,72], and increased NETosis correlates with disease severity, whereas enzymatic clearance of NETs has been shown to induce tissue damage contributing to septic pathology [73]. Dysregulated NETosis has also been implicated in viral infections and a range of autoimmune diseases.

## 9. NETosis Differs Depending on the Polarization of the Neutrophil

To date, research involving neutrophil NETosis has been performed with conflicting results. Some evidence supports oxidized mitochondrial DNA and reactive oxygen species as triggers for NET formation in N1 neutrophils [74]. Specifically, N1 neutrophils have been associated with a robust, anti-tumor, and pro-NETotic state that is called lytic NETosis. N1 neutrophils activate p65 in the NF-κB pathway to upregulate pro-inflammatory cytokines and promote ROS-driven NETosis [75]. N1 neutrophils are characterized by a significantly higher oxidative burst capacity and increased expression of NOX2 subunits [11,76]. High ROS levels are the main driver of lytic NETosis, ensuring the potent release of cytotoxic NETs for pathogen/tumor cell clearance. N1 NETs are often seen as anti-tumor agents due to their cytotoxic components (ROS, neutrophil elastase) and their role in enhancing adaptive immunity [69,76].

In contrast, N2 neutrophils are generally thought to be poised toward a different functional outcome in response to pro-NETotic signals, termed vital NETosis, that may contribute to the creation of a pre-metastatic niche in cancer. TGF-β is the crucial driver of N2 polarization, activating the SMAD-dependent signaling pathway. This pathway regulates genes associated with immune suppression and tissue remodeling, setting the context for pro-tumor NET release. STAT3 and STAT5 are key transcription factors in N2 neutrophils [77]. STAT3 activation enhances the immunosuppressive capacity of neutrophils by upregulating Arginase-1 (Arg-1), which depletes L-arginine and suppresses T-cell function [77]. This pathway also supports N2 survival and differentiation. N2 NETs are implicated in metastasis by forming the pre-metastatic niche and contributing to angiogenesis and immunosuppression, often utilizing a vital NETosis pathway (PAD4-dependent, NOX2-independent) that allows the neutrophil to remain functional [78] (Figure 2).

## 10. Mechanisms of Estradiol-Mediated NETosis

What is the evidence that estradiol triggers NETosis in neutrophils? First, exposing human neutrophils in vitro to pregnancy-level concentrations of human chorionic gonadotropin (hCG) and estradiol triggered robust NET formation [79]. Second, consistent with these findings, analysis of blood serum from pregnant individuals revealed elevated levels of granulocyte colony-stimulating factor (G-CSF), a cytokine known to enhance neutrophil activation and survival, suggesting a broader pro-NETotic milieu during pregnancy. Third, supporting this concept, estradiol treatment induced NETosis in dimethyl sulfoxide (DMSO)-differentiated neutrophil-like HL-60 cells [80].

While evidence indicates that estradiol can trigger NETosis in all neutrophils, estradiol in the context of cancer promotes vital NETosis in N2 neutrophils instead of lytic NETosis in N1 neutrophils. Estradiol promotes NETosis at least in part by stimulating protein arginine deiminase 4 (PAD4) expression, while progesterone may block NET release and the neutrophil is maintained in an extended primed state, highlighting a complex regulatory mechanism [79,80]. This steroidal control may be critical for immune modulation in the female reproductive tract. For example, aberrant NETosis is implicated in pregnancy-related disorders such as preeclampsia (PE), in which the hormonal shift and imbalance may contribute to the dysregulated NET formation [81,82]. In PE, there is an increase in total NETs, including uncitrullinated NETs, which can be an indicator of vital NETosis [83].

Alternatively, several studies have shown that NETosis can be driven not by estrogen stimulation, but rather by estrogen deprivation. Guo et al. (2025) reported that estrogen deficiency (ovariectomy) increased phorbol 12-myristate 13-acetate (PMA)-induced NETosis in bone marrow-derived neutrophils, which in turn polarized bone marrow macrophages toward a pro-inflammatory M1 phenotype and promoted osteoclastogenesis [84]. Mechanistically, this cascade was mediated through activation of the cGAS–STING/AKT2 signaling pathway, linking NET actions to the neutrophil elastase actions on cGAS seen in prostate cancer cells. Pharmacologic inhibition of NETosis with the PAD4 inhibitor GSK484 significantly attenuated osteoporosis in their ovariectomized mouse model [84]. Consistent with these findings, a murine model of intracranial aneurysm (IA) was used to demonstrate that estrogen deficiency led to neutrophil-driven vessel rupture, with excessive NET formation appearing to be the central pathogenic mechanism [85].

Collectively, these studies highlight the unknowns regarding estrogen’s effects on NETosis, demonstrating the need for further studies using various in vitro and in vivo model systems.

## 11. Neutrophil NETs and Estrogen in Epithelial-to-Mesenchymal Transition (EMT)

Neutrophil NETs have been shown to play either pro-tumorigenic or anti-tumorigenic roles in a tissue-specific manner in the context of sex hormone-independent cancers (colorectal and squamous cell carcinoma [86,87]; glioma [88]; and gastric cancer [89]). In contrast, with estrogen-dependent cancers such as breast cancer, NETs have been associated with dangerous hallmarks of metastasis, such as epithelial-to-mesenchymal transition (EMT) [90]. Further, metastasis to the omentum in metastatic ovarian cancer was shown to be dependent on NETs [91].

EMT is a process associated with loss of epithelial cell polarity and development of migratory cell phenotypes [90,92,93]. This mesenchymal morphology contributes to fibrosis and to development of metastasis [94]. In multiple cancers, NETosis has been shown to upregulate genes associated with EMT events, including breast, colorectal, and pancreatic cancers [95]. Following treatment with neutrophil derived NETs, breast cancer cells displayed a mesenchymal morphology and increased migratory capacity [90].

A potential mechanism for EMT induction through NETosis and a NET-sensing receptor may have been discovered. In multiple cancer cell lines, oxidatively damaged NET DNA (8-hydroxyguanosine, or 8-OHG) may act as a ligand for a putative NET cell surface receptor CCDC25 [93]. Further, NET-bound CCDC25 colocalized and interacted with interleukin-linked kinase (ILK) to promote cytoskeletal remodeling in cells [92,93]. In addition to NETosis promoting EMT and metastasis, steroid-sensitive cancer cell proliferation may be enhanced by NET-associated DNA, as mutating CCDC25 diminished NET-induced proliferative increases in several cell lines [93]. Accordingly, bioinformatic analysis of two human cancer databases, the Genotype-Tissue Expression Portal (GTEx) and the Cancer Cell Line Encyclopedia (CCLE), revealed substantially increased expression of CCDC25 NET receptor in breast, lung, prostate, brain, and pancreatic malignant tumor tissues relative to normal tissues [96], with increased CCDC25 expression positively correlated with neutrophil infiltration into tumor tissue.

In summary, the tumor secretome and estradiol appear to work together in attracting neutrophils to the tumor bed, chronically triggering an N2 phenotype that favors vital NETosis. In turn, with the aid of neutrophil elastase, vital NETosis promotes EMT in tumor cells, enhancing migration, invasion, and metastasis, thus promoting overall tumor progression (Figure 2). More studies are needed to fill in the details regarding the pathway, but, nonetheless, studies to date suggest that estradiol signaling as well as NETosis may serve as excellent therapeutic targets in treating aggressive hormone-dependent cancers.

## 12. Ongoing Clinical Trials to Block Neutrophil Actions via NETosis

Clinical trials are underway that involve or directly target NETosis inhibitors, with emphasis on diseases where excessive NET formation is a key driver of pathology, such as autoimmune diseases, thrombosis, and severe inflammation (like acute respiratory distress syndrome (ARDS) or severe COVID-19). Therapeutic strategies generally fall into two categories: NET degraders, which promote breakdown of released NETs, and NET inhibitors, which target enzymes promoting NETosis (such as PADs). PAD inhibitors currently in clinical trials include some specific compounds in the Investigational New Drug (IND) stage, but have not yet entered large-scale Phase I/II trials for NETosis itself [97].

For NET degraders, Dornase alfa (Pulmozyme), already FDA-approved for cystic fibrosis (CF), hydrolyzes extracellular DNA and is currently being evaluated for treatment of other NET-driven diseases such as severe COVID-19-induced pneumonia (https://clinicaltrials.gov/study/NCT01952470, accessed on 21 December 2025) [98]. A second NET-degrading compound, NTR-441, is an albumin-DNASE1L3 fusion protein designed as a plasma protein that will digest extracellular DNA (mainly NETs). This compound has reached human clinical trials. (https://www.clinicaltrials.gov/study/NCT04941183, accessed on 21 December 2025).

Many more NET inhibitors are being investigated in clinical trials than NET degraders. Brensocatib, a neutrophil dipeptidyl peptidase 1 (DPP1) inhibitor, is under evaluation in Phase III clinical trials (also known as the ASPEN study) for bronchiectasis (COPD) [99]. DPP1, or cathepsin C, is a lysosomal enzyme that activates other neutrophil serine proteases (NSPs), including neutrophil elastase. Alvelestat, a NE inhibitor, has been investigated extensively for chronic lung diseases [100] and has passed Phase II trials for bronchiectasis. AZD3241, a myeloperoxidase (MPO) inhibitor, has been studied in the context of neurological disease, using MPO inhibition as a neuroprotective strategy, rather than purely NETosis. It has completed Phase II trials in patients with multiple system atrophy (MSA) to assess microglia activation and suppression of MPO-mediated neuroinflammation (https://clinicaltrials.gov/study/NCT02388295, accessed on 21 December 2025). Another MPO inhibitor, PF-1355, whose clinical trial data is not readily available on public registries, showed promise in animal models in 2016 by attenuating cardiac dilation and inflammation in mice treated with the MPO inhibitor following myocardial infarction and ischemia–reperfusion injury [101].

Notably, most of the above trials are focused on chronic inflammatory conditions rather than cancer, again suggesting the exciting potential for NETosis inhibition as a novel chemotherapeutic area yet to be explored.

## 13. Challenges and Future Directions

### 13.1. Testing Patients for NETosis

An increasing number of diseases involving NETs are coming to light: autoimmune disorders (systemic lupus erythematosus), deep vein thrombosis, chronic viral infections (HIV, COVID-19), neurodegenerative diseases (Alzheimer’s disease), pulmonary diseases (COPD, asthma), and cancers. Despite the rising incidence of NET-related health issues, very few clinicians are aware of NETosis, much less know how to test for NETs. Existing diagnostics for NETosis are highly variable in specificity and the relative difficulty of sample preparation, ranging from plasma smears to identify cell-free DNA and/or NETosing neutrophils to MPO-DNA, NE-DNA, or H3cit-DNA enzyme-linked immunosorbent assays (ELISAs), which are costly, highly specific, and technically rigorous to perform. Notably, these tests will not determine the type of NETosis present (lytic vs. vital) or the tissue of origin, unless a biopsy is performed and analyzed, which is labor-intensive, costly, and invasive. Many barriers exist for implementing regular testing for NETs in the clinical setting, both practical and educational.

### 13.2. Differential Sex-Biased Responses to Cancer Immunotherapy

Females generally respond to immune challenges more robustly than males; however, this can be a double-edged sword. Females will generally have better initial tumor control, but a higher risk of immune-related adverse events from immunotherapies [102]. In a study of cancer patients receiving therapy, women had a higher risk of severe hematologic side effects such as anemia and neutropenia, and increased risk of experiencing 5+ side effects [103]. More research is needed to determine effective immunotherapies for women that do not trigger adverse events, perhaps by limiting ERα and its modulation of NETosis.

### 13.3. Translational Gaps Between Murine Models and Human Tumors

A major reason for the failure of promising preclinical therapies to survive clinical trials in patients is due to translational gaps between mouse and human cancers. Human tumors develop over decades, accumulating a high degree of genetic mutations and heterogeneity both within and between patients. Mouse cancer models typically have a lower mutational burden and are often initiated by one or two engineered driver genes in genetically identical, inbred mice, which results in more homogeneous tumors that do not fully mimic the complexity of human cancer. In addition, mouse models frequently involve xenograft studies conducted in immunodeficient mice with tumor cell lines, which lack a fully intact immune system and thus do not accurately represent complex human tumor interactions with their immune microenvironment. Moreover, the evolution and age of the tumor are quite different between mice and humans: patients usually have a tumor that has evolved through a slow, multi-stage process. In contrast, young (4–8 weeks) immunocompromised mice (nude, SCID, or NSG (scid mutants deficient for functional interleukin-2 receptor subunit gamma that lack mature B, T, and natural killer (NK) cells, and have defective innate immunity)) are given rapidly growing tumorigenic cells that bypass the early tumor establishment process. During the slow generation of human tumors, much more immune training has occurred, which can lead to differential immune–tumor interactions substantially earlier in humans vs. mice.

## 14. Conclusions

The existence of multiple current clinical trials involving neutrophils and NETs, a growing awareness of the involvement of NETosis in a wide range of diseases (including cancer), and the inclusion of neutrophil status in disease prognosis and treatment regimens, points to future co-therapies involving neutrophil and NET suppression. In addition, further elucidation of estradiol’s role in regulating neutrophil production and functions may lead to novel therapies that suppress both direct and neutrophil-mediated effects of estradiol on cancer progression. A specific focus on the relationship between estrogens and NETosis may prove fruitful in suppressing vital NETosis and its pro-tumorigenic effects on the tumor microenvironment with hormone-sensitive tumors. In summary, the study of the estradiol–neutrophil–cancer interactome continues to grow and reveal novel targets that, in the near future, will likely lead to important advances in the treatment of hormone-sensitive and perhaps other cancers.

## Data Availability

No new data were created or analyzed in this study.
